# Dislocation Density in Ceramics Processed by Severe Plastic Deformation via High-Pressure Torsion

**DOI:** 10.3390/ma17246189

**Published:** 2024-12-18

**Authors:** Kaveh Edalati, Nariman Enikeev

**Affiliations:** 1 WPI International Institute for Carbon-Neutral Energy Research (WPI-I2CNER), Kyushu University, Fukuoka 819-0395, Japan; 2 Laboratory of Metals and Alloys Under Extreme Impacts, Ufa University of Science and Technology, 32 Zaki Validi str., Ufa 450076, Russia; nariman.enikeev@gmail.com; 3 Saint Petersburg State Marine Technical University, Lotsmanskaya Str. 10, St. Petersburg 190121, Russia

**Keywords:** severe plastic deformation (SPD), nanostructured ceramics, Rietveld refinement, dislocation density, crystallite size, X-ray diffraction

## Abstract

This study investigates the dislocation density in ceramics processed by severe plastic deformation at room and elevated temperatures via high-pressure torsion (HPT) for various numbers of turns and shear strains. Ceramics, characterized by ionic or covalent bonding, typically exhibit brittleness due to limited dislocation activity. However, HPT enables significant microstructural transformations in ceramics including dislocation nucleation and accumulation. Despite recent advances in the visualization of such dislocations by transmission electron microscopy (TEM), there is a lack of comprehensive reports on the quantification of dislocation density in severely deformed ceramics. This paper addresses this gap by employing X-ray diffraction (XRD) analysis to quantify dislocation density and crystallite size in a few oxide ceramics. Results demonstrate that HPT induces exceptionally high dislocation densities comparable to theoretical upper limits of dislocation density in ceramics, on the order of 10^15^ to 10^16^ m^−2^, with crystallite sizes reduced to the nanometer scale. These findings significantly enhance the understanding of dislocation behavior in ceramics and suggest a potential approach for tuning the mechanical and functional properties of these materials by dislocations.

## 1. Introduction

Ceramics, characterized by their strong ionic and covalent bonds, typically exhibit brittleness at ambient conditions due to limited dislocation activities in their active slip systems [1,2]. Traditional deformation methods for ceramics are based on high-temperature thermally-activated phenomena like creep [3,4] and grain boundary sliding [5,6]. The advent of severe plastic deformation (SPD) techniques [7,8], particularly high-pressure torsion (HPT) [9,10], has opened new avenues for the plastic deformation of ceramics at ambient temperature, enabling significant microstructural transformations [11,12]. HPT is an SPD technique that applies high hydrostatic pressure and torsional straining to materials, inducing substantial plastic deformation without fracturing the material [9,10]. This process is especially beneficial for brittle materials like glasses [13,14] and ceramics [15,16], where traditional deformation methods fall short. The high-pressure environment of HPT suppresses crack propagation, allowing for the maintenance of sample integrity during deformation [14].

The foundations of high-pressure research were laid by Percy W. Bridgman, whose pioneering work in the early 20th century established the principles of applying high pressures to study material behavior [17]. Bridgman’s innovations have been instrumental in developing modern high-pressure techniques, including HPT, which have proven to be highly effective in inducing significant plastic deformation in various materials, including ceramics [10,16]. Motivated by pioneering works by Bridgman [18,19,20] and his successors [21,22,23,24,25,26,27] on HPT processing of ceramics, recent studies have also demonstrated the potential of HPT in processing various traditional ceramics, including oxides [28,29], carbides [14,30], nitrides [31,32] and oxynitrides [33,34]. Moreover, there are applications of HPT to modern ceramics or ceramic-like materials such as silicon [35,36], germanium [37,38], carbon [39,40], hydrides [41,42], and thermoelectric ceramics [43,44]. The HPT-processed ceramics exhibit improved mechanical properties [30,45], enhanced optical properties [46,47], and superior catalytic performance [29,33], among other functional benefits. The improvement of these properties has been shown to be due to unique phase transformations, nanostructured features with significantly refined grain sizes, and large fractions of crystal lattice defects such as vacancies and dislocations [16].

**Figure 1:**
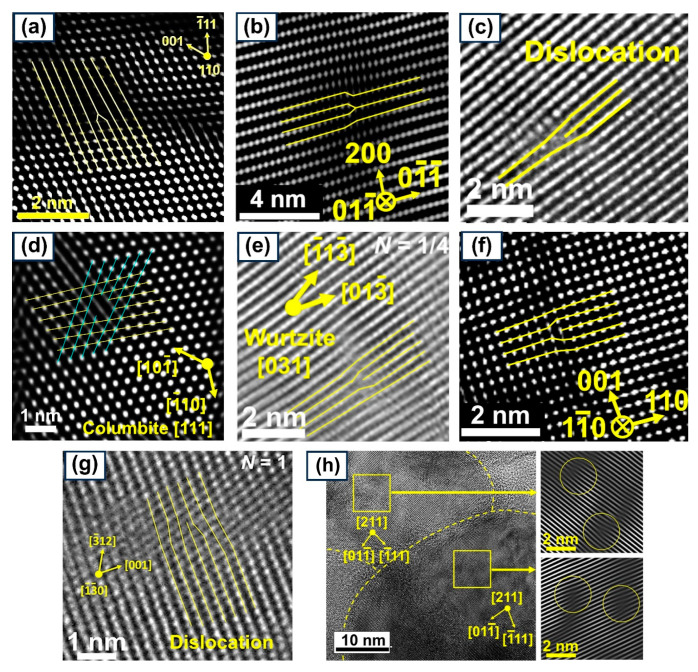
Visualization of dislocations by transmission electron microscopy in HPT-processed ceramics (**a**) MgO [51], (**b**) anatase TiO_2_ [52], (**c**) brookite TiO_2_ [53], (**d**) columbite TiO_2_ [54], (**e**) ZnO [55], (**f**) ZrO_2_ [56], (**g**) BiVO_4_ [57], and (**h**) GaZnON [33].

Despite the advancements in the application of HPT to introduce lattice defects in ceramics, there have been no comprehensive reports on the estimation of the density of these defects in severely deformed ceramics [11,12]. Among various lattice defects, dislocations play a crucial role in the mechanical behavior and functional properties of materials, and their generation and movement are essential for achieving plasticity in ceramics [48,49]. Moreover, dislocations can accelerate phase transformations or lead to the formation of new phases under high pressure [50]. High-resolution transmission electron microscopy (TEM) has been used in previous studies to visualize the formation of dislocations in HPT-processed ceramics, as shown in Figure 1 [33,51,52,53,54,55,56,57]. However, a quantitative analysis of dislocation density remains largely unexplored after HPT processing.

This paper focuses on estimating the dislocation density in ceramics processed by HPT. The aim is to fill the gap in the existing literature by providing a detailed analysis of the dislocation density in these materials using the X-ray diffraction (XRD) analysis. It is shown that the density of dislocations in HPT-processed ceramics can approach the theoretical upper limits of dislocation density in ceramics [58,59] and become as high as those reported in severely deformed metallic materials [60,61]. This represents an important finding that could hardly be anticipated in ceramics due to their ionic or covalent bonding [1,2].

## 2. Materials and Methods

This study primarily utilizes XRD analysis to quantify dislocation density and crystallite size in ceramics processed by HPT. Various XRD-based techniques have been documented in the literature for deriving statistically reliable quantitative data on dislocation density in polycrystalline materials with different crystal symmetries. Although sophisticated methods like convolutional multiple whole profile fitting [62] and whole-powder-pattern modeling [63] offer a comprehensive analysis of diverse dislocation configurations, more straightforward and robust classic procedures are often favored for quickly estimating relative changes in dislocation density. These simpler approaches interpret the microstructural parameters in terms of dislocation density based on several assumptions as will be discussed in detail below. Basic treatment of XRD patterns for such approaches is generally performed using the full profile analysis via the Rietveld refinement method [64,65].

### 2.1. Sample Preparation

Samples of ceramic powders, including typical oxides MgO (99.99% from Kojundo, Sakado, Japan), α-Al_2_O_3_ (99.99% from Kojundo, Japan), γ-Al_2_O_3_ (99.9% from Kojundo, Japan), ZnO (99.5% from Kanto Chemicals, Tokyo, Japan) and BiVO_4_ (99.9% from Alfa Aesar, Lancashire, UK) were subjected to HPT under different strain conditions. These five oxide ceramics were selected as representative ceramics with diverse crystal structures and a wide range of mechanical and functional applications such as load-bearing parts, solar cells, photocatalysts, and so on. An appropriate number of samples (300 mg for MgO, 500 mg for Al_2_O_3_, 400 mg for ZnO, and 410 mg for BiVO_4_) were compacted to discs with 10 mm diameter using a manual press under a pressure of 0.38 GPa. The compacted discs were processed by HPT under a pressure of 6 GPa and a rotation speed of 1 rpm using a pair of anvils made from a WC-11Co (wt%) composite. The shear strain (*γ* = 2 π*rN*/*h*; *γ*: shear strain; *r*: distance from the rotation axis, *N*: HPT turns, *h*: material thickness) was introduced by applying rotation numbers of *N* = 0 (pure compression), 1/16, 1/4, 1, and 4 at various temperatures of *T* = 300, 473, 573, 673, and 723 K (not all samples were processed under all these conditions). It should be noted that a temperature rise usually occurs during HPT processing at room temperature, but its level was not significant in this study to affect the microstructural evolution (<20 K measured by a thermocouple located in the upper anvil of HPT and by an optical thermometer). This insignificant temperature rise, which is in good agreement with an earlier study using experiments and finite element simulation [66], is due to the acting of massive HPT anvils as heat sinks. The samples after HPT, which were in the form of compacted discs with dark colors, were crushed using a mortar and pestle for XRD analysis. Detailed structural and microstructural evolution for these ceramics during HPT are given in the literature for MgO [51], α-Al_2_O_3_ [45], γ-Al_2_O_3_ [67,68], ZnO [55], and BiVO_4_ [57].

### 2.2. X-Ray Diffraction

XRD measurements were performed using a Rigaku SmartLab diffractometer (Tokyo, Japan) with Cu *Kα* radiation at 45 kV and 40 mA with 0.05° scanning step and of 2°/min scanning speed. The diffraction patterns were recorded over a 2*θ* range of 10° to 100°.

### 2.3. Dislocation Density Calculation

The full-profile analysis by the Rietveld refinement technique using the MAUD software ver. 2.9995 [69] was used to obtain values for lattice parameters (Table 1), crystallite size and microstrain. To ensure reasonable reproduction of the XRD data by Rietveld refinement, parameters of fitting quality were traced. Among the parameters, weighted profile *R*-factor (*R*_wp_) and expected *R*-factor (*R*_exp_) were kept below 20%. Moreover, the so-called Chi-squared (χ^2^ = (*R*_wp_/*R*_exp_)^2^) or goodness of fit (*G* = χ) was checked to approach unity but kept above. While XRD peak position is determined by lattice parameters, XRD peak broadening is affected by dislocations, crystallite size, vacancy clusters and instrumental peak broadening (caused by the X-ray source and the measurement system). The instrumental peak broadening was accounted for by a specialized treatment of the profile of a standard silicon sample. The influence of vacancies on peak broadening was ignored as proposed in an earlier study [70], and the remaining effect of crystallite size and dislocation density was considered as described below.

It is still a matter of discussion which is the most appropriate approach to estimate all strain-induced dislocations in deformed polycrystalline materials. The interpretation of XRD-deduced parameters in terms of dislocation density largely depends on microstructural features of the investigated materials and specific distributions of dislocations induced by straining. In fact, the classic approach proposed in the middle of the last century to calculate dislocation densities [71] shall be differently applied to annealed and severely deformed materials, while it also depends on strain and dislocation distributions. Assuming a block structure with boundaries formed by dislocations in cold-worked metals, a classic Equation (1) from [71] is written as follows.

(1) $$\rho_{p}=\frac{3n}{D^{2}}$$

where *D* is an XRD-measured particle/block size with *n* dislocations per block face (*n* = 1 for severely deformed metals [71]) and *ρ*_p_ is the full dislocation density. In this formula, *ρ*_p_ is the total length of dislocation lines per unit volume, not depending on the distribution of dislocations. If an isotropic (orientation-independent) distribution of dislocations is assumed, then another expression can be proposed from [71], based on considerations put forth in [72].

(2) $$\rho_{p}=\frac{n}{D^{2}}$$

The dislocation density can also be estimated from the XRD-measured microstrain value according to [71].

(3) $$\rho_{s}=\frac{k}{F}\frac{\varepsilon^{2}}{b^{2}}$$

where *ρ*_s_ is the dislocation density, *b* is the Burgers vector value, *F* accounts for dislocation interaction, *ε*^2^ is XRD-measured microstrain and *k* = 12*A*, where *A* depends on the shape of strain distribution.

The total dislocation density *ρ* at the extreme degree of dislocation interaction can be achieved via the following expression [71].

(4) $$\rho =\sqrt{\rho_{s}\rho_{p}}$$

The absolute value of *ρ* can considerably depend on the distribution of dislocations and strain. For example, *A* can vary from 1/(2π) to 2 for Gaussian and Cauchy distributions, respectively, which means a range for *k* of 1.9 to 24. This indicates that the values of *ρ* may differ by almost an order of magnitude depending on the assumptions. Here, it should be noted that some researchers might misinterpret the variation in *A* magnitude because it is not obvious in [71] that the lower value for *A* is 1/(2*π*), while the range for *k* values has been clearly specified as 2 to 25. This issue was clarified in [73], where *A* for the Gaussian distribution was explicitly specified as 1/(2π). Hence, it is natural to follow the assumption proposed in [73] and assign *A* to unity as an intermediate value, which brings *k* = 12. The validity of such an approach was confirmed by calorimetric measurements and estimations of stored energy [73]. With all these assumptions, a combination of Equations (3) and (4) leads to the following relationship.

(5) $$\rho =\sqrt{\frac{k}{F}\frac{\varepsilon^{2}}{b^{2}}\rho_{p}}$$

With *k* = 12, *F* = *n* [71] and substituting *ρ*_p_ with Equation (1), the following relationship is achieved.

(6) $$\rho =\frac{6<\varepsilon^{2}>}{bD}$$

while with *ρ_p_* taken from Equation (2), another equation is achieved.

(7) $$\rho =\frac{2\sqrt{3}<\varepsilon^{2}>}{bD}$$

Early implementations of approaches based on Equations (1)–(4) [71,73] were made in controversial ways. As mentioned above, several following studies proposed to use *k* = 6π with *A =* π/2 instead of *A* = 1/(2π) for the case of purely Gaussian strain distributions. For example, in [74,75], Equation (4) was used with *k* = 18.8 or *k* = 6*π* and *ρ*_p_ taken from Equation (1), which sometimes is reproduced nowadays [76]. Authors of [77] used Equations (2) and (3) with *k* = 12 and reported its merit when measuring dislocation density becomes complicated with using TEM. Equation (7) is considered to be appropriate for the evaluation of SPD-processed nanostructured materials. Early XRD studies applied to SPD nanomaterials explicitly used Equation (7) to evaluate the dislocation density in copper subjected to HPT [78], copper subjected to equal-channel angular pressing [79], ball-milled nickel nanopowders consolidated by HPT [79] and copper and palladium processed by inert gas condensation followed by HPT consolidation [79]. After these early publications, Equation (7) has become very popular among researchers looking for a quick and robust XRD-based approach to evaluate the dislocation density in SPD-processed metallic materials with cubic crystal structure. In principle, Equation (6) better suits the original ideas described in [71,73]; however, Equation (7) together with Equation (2) are used in this study for ceramics with cubic structure to keep consistency with the reports about SPD-processed metals. It should be noted that since Equation (2) underestimates the total dislocation density [71,72] due to possible stress screening of dislocations of opposite signs [72], Equations (1) and (6) might are other options that can be tested for polycrystalline materials in future studies.

Equation (7) is straightforward for cubic systems characterized by a single Burgers vector. For materials with non-cubic lattices, correct estimating dislocation density is much more complicated due to the presence of multiple Burgers vectors. For example, in order to characterize dislocation densities in hexagonal crystals, like hexagonal close-packed (HCP), approaches such as the one proposed by Griffiths et al. [80] can be employed. It is considered that HCP polycrystals are plastically deformed by slip of <*a*> and <*c+a*> dislocations followed by generation of <*c*>-type dislocations as a result of dislocation interactions. Different dislocation types can be resolved to their *a* and *c* components which would differently contribute to the broadening of X-ray peaks belonging to the basal and prismatic planes [80]. Accordingly, using deconvolution peak profile analysis to calculate the corresponding size and microstrain contributions for each peak family, one may assess the density of dislocations with respect to their *a* and *c* components as given below [80].

(8) $$\rho_{a}=\frac{K_{a}\varepsilon_{a}^{2}}{b_{a}^{2}}/\ln\frac{d_{a}}{2r_{0}}$$

(9) $$\rho_{c}=\frac{K_{c}\varepsilon_{c}^{2}}{b_{c}^{2}}/\ln\frac{d_{c}}{2r_{0}}$$

In these two equations, *K*_a_ = 52.1, *K*_c_ = 26.1, and *r*_0_ = 1 nm [80]. The values of Burgers vectors for dislocations of *a* and *c* components are denoted as *b*_a_ and *b*_c_, where *b*_a_ is calculated as $1/3<11\bar{2}0>$ and *b*_c_ as [0001]. Crystallite sizes and microstrains calculated for the prism crystallographic planes $\left\{10\bar{1}0\right\}$ and $\left\{11\bar{2}0\right\}$ are denoted as *d*_a_ and *ε_a_* and for (0001) basal planes as *d*_c_ and *ε_c_*, respectively [80]. These values are deduced from experimental XRD profiles by application of the anisotropic Popa line broadening model [81] during the Rietveld refinement procedure to account for different broadening contributions in reflections from various crystal planes. Although there are successes in the application of this combined approach to severely deformed metallic materials with HCP symmetry [82,83], the applicability of this method for hexagonal ceramics has not been examined yet.

For materials with symmetries other than cubic and hexagonal, many studies use a general Equation (2) for dislocation density. This formula assumes that the entire peak broadening is induced by the size effect, which can be a practical approach for deformed ceramics with complex crystal symmetries. It should be noted that Equation (2) and other approaches mentioned above are based on serious approximations and cannot be considered rigorous. An in-depth analysis in [84] critically discussed the validity of Equation (7), especially when combined with simplified size–strain deconvolution techniques. Even if the absolute values of dislocation density can hardly be claimed (similar to any other technique), the trends in evolving dislocation can safely be considered reliable using a uniform approach.

According to the assumptions for choosing an approach for calculating dislocation densities, the required parameters were achieved by XRD analysis depending on the crystal symmetry of the materials (Table 1). For cubic symmetry, the Rietveld refinement method with the isotropic model for size–strain deconvolution was used. Then the calculated values of crystallite sizes and microstrains were used in Equation (7). For ceramics with a hexagonal crystal structure, the Rietveld method was realized by refining the parameters of the Popa model of anisotropic line broadening, achieving crystallite sizes and microstrains for different plane families. Then, the values corresponding to prism planes were used in Equation (8) together with Burgers vector value for <*a*>-type dislocations. In the generalized case of materials with lower symmetries, size and strain effects were not separated and strain broadening was fixed at the negligible level so that the whole peak broadening was interpreted as the size effect. The obtained values were then used in Equation (2) with *n* = 1 as for very severely deformed materials [71].

## 3. Results

**Figure 2:**
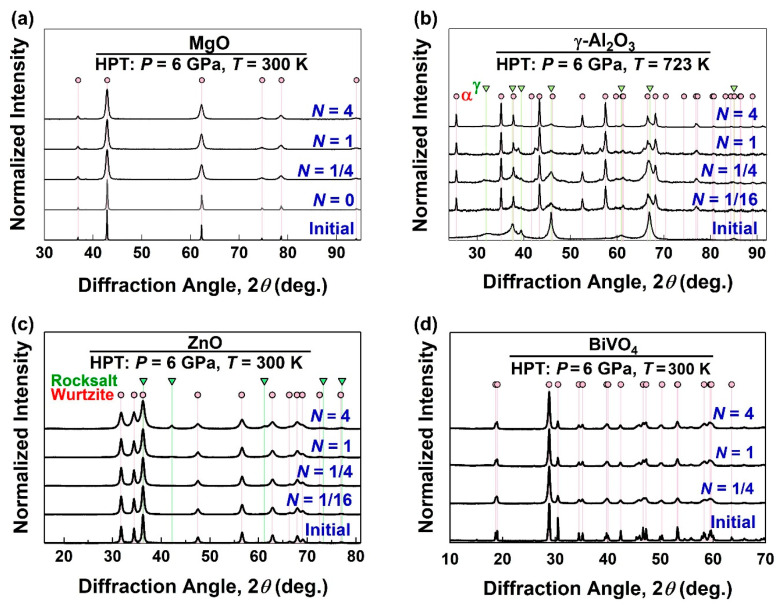
XRD profiles before and after various HPT turns for ceramics (**a**) MgO processed at room temperature, (**b**) γ-Al_2_O_3_ processed at 723 K, (**c**) ZnO processed at room temperature, and (**d**) BiVO_4_ processed at room temperature.

**Figure 3:**
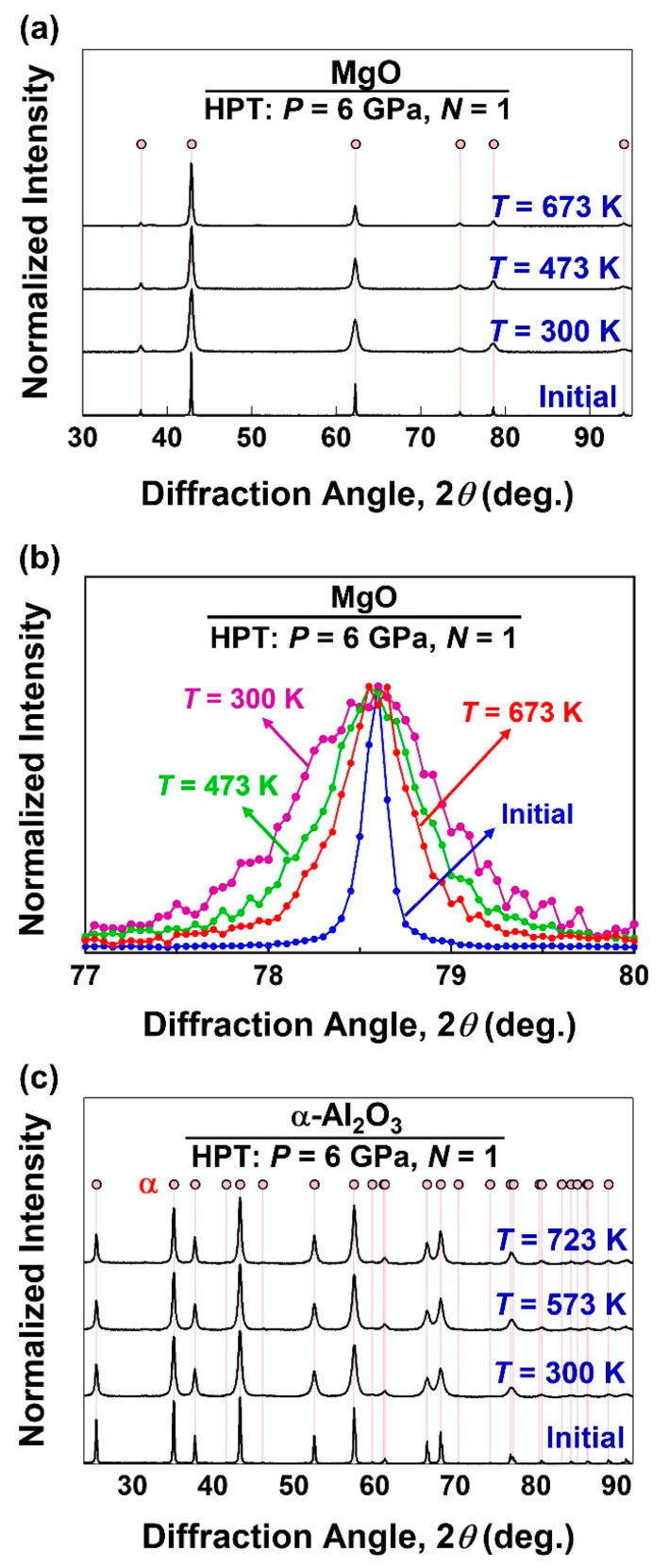
XRD profiles of (**a**,**b**) MgO and (**c**) α-Al_2_O_3_ processed by HPT for *N* = 1 turn at various temperatures, where (**b**) is a magnified view of (222) peak of MgO in (**a**).

The XRD patterns of the HPT-processed ceramics before and after various HPT turns are shown in Figure 2 for (a) MgO processed at room temperature, (b) γ-Al_2_O_3_ processed at 723 K, (c) MgO processed at room temperature, and (d) BiVO_4_ processed at room temperature. While MgO and BiVO_4_ show only peak broadening after HPT processing without any phase transformations, metastable γ-Al_2_O_3_ transforms to a stable α phase by HPT processing. ZnO also exhibits a phase transformation to a small amount of a high-pressure rocksalt phase. It should be noted that none of the oxides studied in this study exhibit amorphization by HPT treatment. Figure 3 illustrates the XRD profiles of (a, b) MgO and (c) α-Al_2_O_3_ processed for *N* = 1 turns at various temperatures where (b) is a magnified view of (a) to show peak broadening changes. For both ceramics, a peak broadening occurs after HPT processing, but it becomes less significant when the HPT processing temperature is increased. XRD peak broadening is an indication of lattice strain due to the generation of defects and crystallite size reduction [64,65,72]. Such peak broadening has been frequently reported in a wide range of HPT-processed metallic materials [9,11,12] and ceramics [28,29,47]. While there have been numerous studies on using such peak broadening to quantify the dislocation density in severely deformed metals [60,61,82,83], this quantification approach for SPD-processed ceramics is missing in the literature and is reported below.

**Figure 4:**
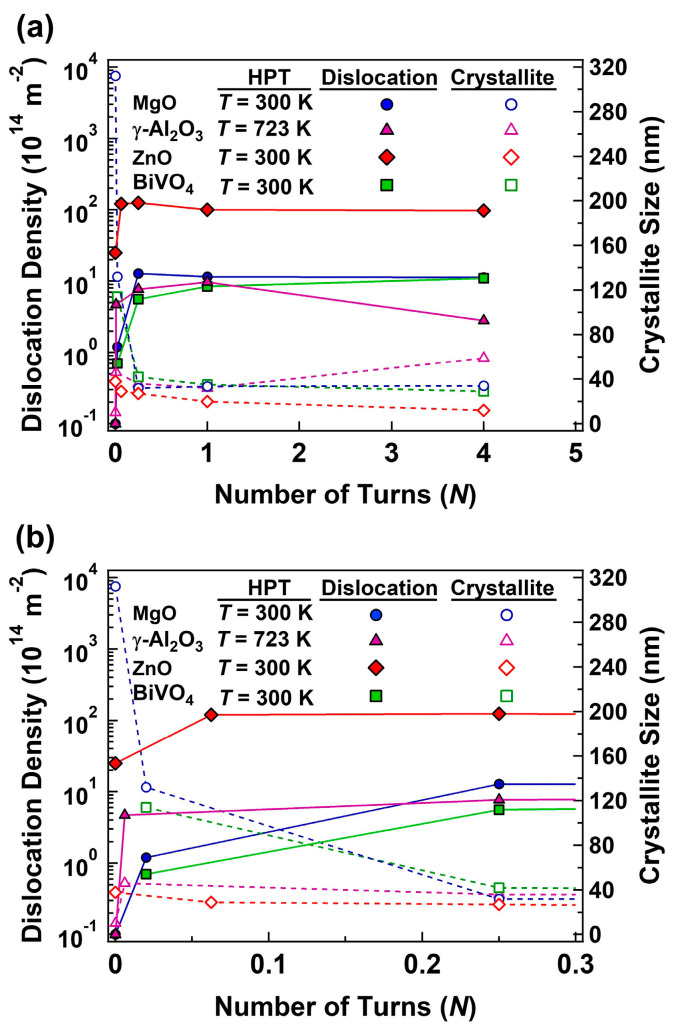
Variations in dislocation density and crystallite size versus the number of HPT turns for MgO processed at room temperature, γ-Al_2_O_3_ processed at 723 K, ZnO processed at room temperature, and BiVO_4_ processed at room temperature, where (**b**) is a magnified view of (**a**) at low number of turns.

Figure 4 shows the variations in dislocation density and crystallite sizes versus the number of HPT turns for MgO, γ-Al_2_O_3_, ZnO, and BiVO_4_. The XRD analysis reveals a substantial decrease in crystallite size, from the submicrometer level in the as-received MgO and BiVO_4_ powders to nanometer-scale dimensions after HPT processing. The results for these two ceramics show that HPT processing leads to a dramatic increase in dislocation density, reaching values on the order of 10^15^ m^−2^. For ZnO, the dislocation density reaches the order of 10^16^ m^−2^ which is higher than those achieved in MgO and BiVO_4_, perhaps due to the formation of the rocksalt phase and its effect on pinning the dislocations. For γ-Al_2_O_3_, there is also an increase in dislocation density after HPT processing, but crystallite size increases due to a structural transition to the α phase [67,68]. These high dislocation densities achieved in this study are comparable to those observed in severely deformed metals [60,61,82,83], underscoring the effectiveness of HPT in generating a high density of dislocations even in hard and brittle ceramics at room temperature. In good agreement with these calculations, earlier TEM observations indicated the localized density of dislocations in the order of 10^15^ m^−2^ or higher in some HPT-processed ceramics (e.g., 1 *×* 10^15^ m^−2^ in GaZnON [33], 2 *×* 10^15^ m^−2^ in TiO_2_ [52], 2 *×* 10^15^ m^−2^ in ZrO_2_) [56]. Such high densities are also consistent with a theoretically predicted upper limit of dislocation density in single-phase ceramics [59] and are achieved in some rare cases such as ball-milled high-entropy ceramics [58]. Here, it should be noted that crystallite sizes measured in this study (34 nm for MgO, 59 nm for Al_2_O_3_, 12 nm for ZnO and 29 nm for BiVO_4_) are reasonably consistent with grain sizes reported using TEM in the literature (>10 nm for MgO [51], 10–200 nm for Al_2_O_3_ [67,68], 9 nm for ZnO [55], 11 ± 5 nm for ZnO [85] and 15 nm for BiVO_4_ [57]), indicating the reliability of current XRD analyses.

**Figure 5:**
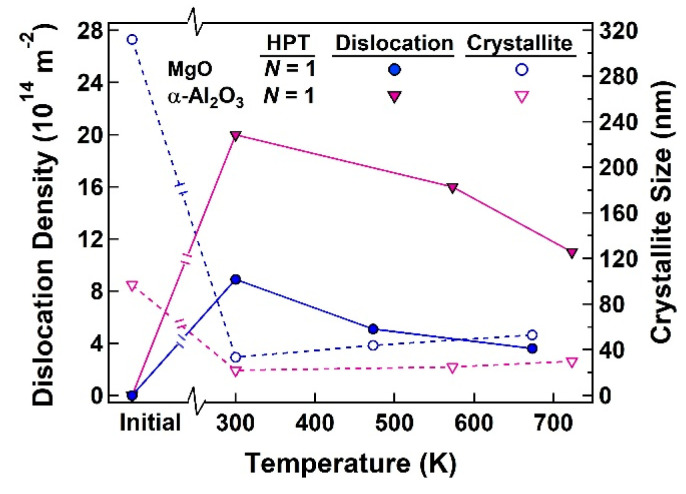
Variations in dislocation density and crystallite sizes versus HPT processing temperature for MgO and α-Al_2_O_3_ processed by HPT for *N* = 1 turn.

Figure 5 shows the variations in dislocation density and crystallite size versus the HPT processing temperature for MgO and α-Al_2_O_3_ processed for *N* = 1 turn. For both ceramics, dislocation density increases after HPT processing at room temperature, but decreases with increasing the processing temperature. There is also an increase in the dislocation density, but the increases become less significant with elevating processing temperature. The effect of temperature on the crystallite size and dislocation density should be due to the thermal enhancement of atomic mobility and the promotion of grain boundary migration and dislocation annihilation as reported in metallic materials [9,86] and discussed below.

A combination of Figure 4 and Figure 5 shows that the dislocation density increases and the crystallite size decreases with increasing the number of HPT turns, while such changes become less significant with increasing the HPT processing temperature. As in metallic materials, severe straining by increasing the number of HPT turns provides an accumulation of dislocations in ceramics, and hence the dislocation density increases. Alongside that, the intense interaction of dislocations results in reducing inter-dislocation distances and also leads to grain refinement [11,12]. On the other hand, increasing the HPT processing temperature enhances the diffusion-controlled mechanisms of dislocation recovery and annihilation as well as grain boundary migration and recrystallization [11,12]. Since the processes of recovery, recrystallization, and grain boundary migration compete with strain-induced dislocation multiplication and accumulation processes, high processing temperature leads to a reduction in dislocation density and an increase in crystallite size as shown in Figure 5. This behavior is fully consistent with the trends observed for the numerous metals and alloys subjected to SPD [7,8,9,10,11,12,86].

## 4. Discussion

The generation of dislocations in ceramics through HPT is facilitated by severe plastic strain imposed by the process [7,8,9,10]. The high-pressure environment inherent in HPT plays a crucial role by inhibiting crack propagation [13,14,15], which typically limits plastic deformation in ceramics due to their covalent or ionic bonding nature and limited active slip systems [1,2,3,4,5,6]. This environment allows for extensive dislocation activity, even in materials that are traditionally brittle [11,12]. The dislocation densities reported in this study are remarkably high for ceramics [58,59], which underscores the effectiveness of HPT in altering the microstructure of ceramics. High-resolution TEM images from previous studies provide further context for these findings, revealing dense networks of dislocations after HPT processing [33,51,52,53,54,55,56,57]. Although the dislocation density was examined for five representative ceramics, the chosen approach in this study is universal and can be applied to any type of crystalline ceramics and, in general, to any deformed polycrystalline materials, by considering their crystal symmetry and dislocation structure. Here, two key issues are discussed: (i) the significance of such high dislocation densities, and (ii) the similarities and differences in the evolution of dislocations and microstructure under strain in ceramics versus metals.

### 4.1. Significance of High Dislocation Density in Ceramics

One of the primary challenges in enhancing the plasticity of ceramics is the difficulty associated with dislocation nucleation [1,2]. Recent studies suggest that ceramics can exhibit plasticity if dislocations are nucleated from an external source, such as metal-ceramic interfaces [2]. However, HPT processing demonstrates that dislocations can be generated internally, without the need for external nucleation sites, until the dislocation density reaches the upper theoretical limits [59]. Such high dislocation density is expected to contribute significantly to the strength of ceramics and could also enhance their plasticity [1,2]. This is a critical finding, as it opens up new possibilities for developing ceramics with improved mechanical properties and enhanced functionality. Future research focused on the strength-plasticity combination in HPT-processed ceramics could further elucidate the potential of these nanostructured materials.

Dislocations also play a critical role in strain- and pressure-induced phase transformations in ceramics [36,56]. Theoretical studies have proposed that a strong stress field at the tip of dislocation pileups under high pressure can accelerate strain-induced phase transformations and decrease the phase transition pressure [87]. The data presented in this study support these theoretical predictions, indicating that HPT not only enhances dislocation density but also potentially influences phase stability and transformation behavior in ceramics through dislocation activity. It should be noted acceleration of phase transformation by HPT has been a hot research topic for decades [17,18,19,20,21,22,23,24,25,26,27]. It is well established that a wide range of ceramics or ceramic-like materials, including oxides [28,29,73,88], carbides [14], nitrides [31,32], silicon [35,36], germanium [37,38], carbon [39,40], and hydrides [41,42], can exhibit accelerated phase transformations by HPT processing.

### 4.2. Comparison of Dislocation and Microstructure Evolution in Ceramics and Metals

This study indicates that dislocation densities increase and crystallite sizes decrease with the number of HPT turns, i.e., with increasing applied shear strain. However, both parameters eventually reach steady-state values at large shear strains, a behavior that closely mirrors what is observed in metals [7,8,9,10,11,12]. The occurrence of a steady state in ceramics, as in metals, likely results from a balance between dislocation generation and annihilation through dynamic recovery [60], as well as a balance between grain refinement and growth through dynamic recrystallization and grain boundary migration [86]. This consideration is consistent with the results achieved for the ceramics, processed at elevated temperatures (Figure 4), where dislocation density in Al_2_O_3_ deformed at 723 K tends to reduce when strain increases by HPT processing to a higher number of rotations.

However, a significant difference between metals and ceramics is that the steady-state crystal sizes in HPT-processed ceramics are about one order of magnitude smaller than those reported in metals [7,8,9,10,11,12]. Additionally, the onset of a steady state in ceramic materials occurs at much lower strains compared to metallic materials [86,89]. This smaller steady-state grain size in ceramics is likely due to difficult dynamic recrystallization, a consequence of their ionic/covalent bonding and high melting points [16]. Before reaching the steady state, recrystallization and recovery in ceramics are minimal, which may explain why the microstructure evolves more rapidly in ceramics than in metals.

Although one might expect a higher dislocation density in ceramics due to the difficulty in dislocation mobility and recovery, the dislocation densities measured in this study are comparable to those reported in metals [60]. This could be attributed to the small crystallite size of HPT-processed ceramics, which limits the number of dislocations within the grain interiors because grain boundaries in nanograined materials can act as dislocation sinks [90]. Consequently, while the microstructural evolution in ceramics and metals during HPT follows similar mechanisms, involving dislocation nucleation, dislocation accumulation (either as subgrain boundaries or dislocation pileups), dynamic recrystallization, and the formation of high-angle grain boundaries, and finally saturation of microstructural evolution to the steady state [7,9], the outcomes differ significantly in terms of grain size but are similar in terms of dislocation density. Future research in this field should focus on (i) coupled TEM and XRD analysis of dislocation density, as some studies raised severe concerns regarding the use of XRD alone for the estimation of dislocation density [91], (ii) dislocation density analyses on non-oxide HPT-processed ceramics such as silicon [35,36,50], germanium [37,38], carbon [39,40], hydrides [41,42] and thermoelectric ceramics [43,44], and (iii) examination of the mechanical properties (e.g., hardness, fracture toughness, plasticity, damage tolerance) of ceramics with such high dislocation densities. It should be noted that ceramic powders processed by HPT transform into compacted powders and not dense bulk discs [92], and thus, to achieve bulk samples for mechanical property test, an additional sintering process is needed, which can reduce the dislocation density [28,30,45].

## 5. Conclusions

This study demonstrates the efficacy of severe plastic deformation via high-pressure torsion (HPT) in inducing significant dislocation nucleation and activity in ceramics, resulting in a high dislocation density. The use of X-ray diffraction (XRD) analysis provides a robust method for quantifying dislocation density and crystallite size, offering valuable insights into the microstructural evolution of HPT-processed ceramics. The high steady-state dislocation density achieved through HPT processing was in the range of 10^15^ to 10^16^ m^−2^ which is close to the theoretical upper limit of dislocation density in ceramics. These findings significantly advance the understanding of dislocation behavior in ceramics at ambient and low temperatures, highlighting the potential of HPT for the development of ceramics with tailored properties for mechanical and functional applications. Future work should focus on exploring the relationship between these microstructural changes and the resulting properties to fully exploit the benefits of HPT processing in ceramic materials.

## Figures and Tables

**Table 1:** Lattice parameters, Burgers vector and formula used for the calculation of dislocation density for different ceramics in this study.

Ceramics	Lattice Parameters (Å)	Burgers Vector	Equation Used for Dislocation Density
MgO	*a* = 4.212 ± 0.0002	$a\sqrt{2}/2$	(7)
ZnO	*a* = 3.251 ± 0.0001 *b* = 5.210 ± 0.0002	*a* for *a* component	(8)
BiVO_4_	*a* = 5.195 ± 0.0008 *b* = 5.091 ± 0.0007 *c* = 11.701 ± 0.0017	---	(2)
α-Al_2_O_3_	*a* = 4.757 ± 0.0002 *b* = 12.983 ± 0.0006	---	(2)
γ-Al_2_O_3_	*a* = 7.915 ± 0.0032	---	(2)

## Data Availability

The original contributions presented in this study are included in the article. Further inquiries can be directed to the corresponding author.

## References

[1] Porz L. (2022). 60 years of dislocations in ceramics: A conceptual framework for dislocation mechanics in ceramics. Int. J. Ceram. Eng. Sci..

[2] Mitchell T.E., Lagerlöf K.P.D., Heuer A.H. (1985). Dislocations in ceramics. Mater. Sci. Technol..

[3] Cannon W.R., Langdon T.G. (1983). Creep of ceramics: Part 1 mechanical characteristics. J. Mater. Sci..

[4] Cannon W.R., Langdon T.G. (1988). Creep of ceramics: Part 2. an examination of flow mechanisms. J. Mater. Sci..

[5] Wakai F. (1986). Superplasticity of yttria-stabilized tetragonal ZrO_2_ polycrystals. Adv. Ceram. Mater..

[6] Kim B.N., Hiraga K., Morita K., Sakka Y., Yamada T. (2002). Enhanced tensile ductility in ZrO_2_-Al_2_O_3_-spinel composite ceramic. Scr. Mater..

[7] Valiev R.Z., Islamgaliev R.K., Alexandrov I.V. (2000). Bulk nanostructured materials from severe plastic deformation. Prog. Mater. Sci..

[8] Valiev R.Z., Estrin Y., Horita Z., Langdon T.G., Zehetbauer M.J., Zhu Y.T. (2016). Producing bulk ultrafine-grained materials by severe plastic deformation: Ten years later. JOM.

[9] Zhilyaev A.P., Langdon T.G. (2008). Using high-pressure torsion for metal processing: Fundamentals and applications. Prog. Mater. Sci..

[10] Edalati K., Horita Z. (2016). A review on high-pressure torsion (HPT) from 1935 to 1988. Mater. Sci. Eng. A.

[11] Edalati K., Bachmaier A., Beloshenko V.A., Beygelzimer Y., Blank V.D., Botta W.J., Bryła K., Čížek J., Divinski S., Enikeev N.A. (2022). Nanomaterials by severe plastic deformation: Review of historical developments and recent advances. Mater. Res. Lett..

[12] Edalati K., Ahmed A.Q., Akrami S., Ameyama K., Aptukov V., Asfandiyarov R.N., Ashida M., Astanin V., Bachmaier A., Beloshenko V. (2024). Severe plastic deformation for producing superfunctional ultrafine-grained and heterostructured materials: An interdisciplinary review. J. Alloys Compd..

[13] Edalati K., Yokoyama Y., Horita Z. (2010). High-pressure torsion of machining chips and bulk discs of amorphous Zr_50_Cu_30_Al_10_Ni_10_. Mater. Trans..

[14] Levitas V.I., Ma Y., Selvi E., Wu J., Patten J. (2012). High-density amorphous phase of silicon carbide obtained under large plastic shear and high pressure. Phys. Rev. B.

[15] Greenberg B.A., Ivanov M.A., Pilyugin V.P., Pushkin M.S., Plotnikov A.V., Tolmachev T.P., Patselov A.M., Tankeyev A.P. (2018). Microstructural evolution in ceramics and glasses during high pressure torsion. Russ. Metall..

[16] Edalati K. (2019). Review on recent advancements in severe plastic deformation of oxides by high-pressure torsion (HPT). Adv. Eng. Mater..

[17] Bridgman P.W. (1935). Effects of high shearing stress combined with high hydrostatic pressure. Phys. Rev..

[18] Bridgman P.W. (1937). Shearing phenomena at high pressures, particularly in inorganic compounds. Proc. Am. Acad. Arts Sci..

[19] Larsen E.S., Bridgman P.W. (1938). Shearing experiments on some selected minerals and mineral combinations. Am. J. Sci..

[20] Bridgman P.W. (1936). Shearing phenomena at high pressure of possible importance for geology. J. Geol..

[21] Griggs D.T. (1937). Deformation of rocks under high confining pressure. J. Geol..

[22] Bates C.H., White W.B., Roy R. (1963). New high-pressure polymorph of zinc oxide. Science.

[23] Bell P.M. (1963). Aluminum silicate system: Experimental determination of the triple point. Science.

[24] Dachille F., Roy R. (1964). Effectiveness of shearing stresses in accelerating solid-phase reactions at low temperatures and high pressures. J. Geol..

[25] Vereshchagin L.F., Zubova E.V., Burdina K.P., Aparnikov G.L. (1971). Behaviour of oxides under the action of high pressure with simultaneous application of shear stresses. Dokl. Akad. Nauk. SSSR.

[26] Morozova O.S., Maksimov Y.V., Shashkin D.P., Shirjaev P.A., Matveyev V.V., Zhorin V.A., Krylov O.V., Krjukova G.N. (1991). Carbon monoxide hydrogenation over iron oxide, subjected to shear deformation under high pressure: Role of vacancies. Appl. Catal..

[27] Blank V.D., Konyaev Y.S., Kuznetsov A.I., Estrin E.I. (1984). Diamond chamber for examining the effects of shear deformation on the structure and properties of solids at pressures up to 43 GPa. Instrum. Exp. Tech..

[28] Edalati K., Arimura M., Ikoma Y., Daio T., Miyata M., Smith D.J., Horita Z. (2015). Plastic deformation of BaTiO_3_ ceramics by high-pressure torsion and changes in phase transformations, optical and dielectric properties. Mater. Res. Lett..

[29] Wang Q., Edalati K., Koganemaru Y., Nakamura S., Watanabe M., Ishihara T., Horita Z. (2020). Photocatalytic hydrogen generation on low-bandgap black zirconia (ZrO_2_) produced by high-pressure torsion. J. Mater. Chem. A.

[30] Edalati K., Iwaoka H., Toh S., Sasaki K., Horita Z. (2013). Application of high-pressure torsion to WC-Co ceramic-based composites for improvement of consolidation, microstructure and hardness. Mater. Trans..

[31] Levitas V.I., Shvedov L.K. (2002). Low pressure phase transformation from rhombohedral to cubic BN: Experiment and theory. Phys. Rev. B.

[32] Ji C., Levitas V.I., Zhu H., Chaudhuri J., Marathe A., Ma Y. (2012). Shear-induced phase transition of nanocrystalline hexagonal boron nitride to wurtzitic structure at room temperature and low pressure. Proc Natl. Acad. Sci. USA.

[33] Edalati K., Uehiro R., Takechi S., Wang Q., Arita M., Watanabe M., Ishihara T., Horita Z. (2020). Enhanced photocatalytic hydrogen production on GaN-ZnO oxynitride by introduction of strain-induced nitrogen vacancy complexes. Acta Mater..

[34] Akrami S., Edalati P., Shundo Y., Watanabe M., Ishihara T., Fuji M., Edalati K. (2022). Significant CO_2_ photoreduction on a high-entropy oxynitride. J. Chem. Eng..

[35] Ikoma Y., Hayano K., Edalati K., Saito K., Guo Q., Horita Z. (2012). Phase transformation and nanograin refinement of silicon by processing through high-pressure torsion. Appl. Phys. Lett..

[36] Chen H., Levitas V.I., Xiong L. (2019). Amorphization induced by 60 shuffle dislocation pileup against different grain boundaries in silicon bicrystal under shear. Acta Mater..

[37] Edalati K., Horita Z. (2011). Correlations between hardness and atomic bond parameters of pure metals and semi-metals after processing by high-pressure torsion. Scr. Mater..

[38] Blank V.D., Popov M.Y., Kulnitskiy B.A. (2019). The effect of severe plastic deformations on phase transitions and structure of solids. Mater. Trans..

[39] Blank V.D., Popov M., Buga S.G., Davydov V., Denisov V.N., Ivlev A.N., Marvin B.N., Agafonov V., Ceolin R., Szwarc H. (1994). Is C60 fullerite harder than diamond. Phys. Lett. A.

[40] Edalati K., Daio T., Ikoma Y., Arita M., Horita Z. (2013). Graphite to diamond-like carbon phase transformation by high-pressure torsion. Appl. Phys. Lett..

[41] Edalati K., Kitabayashi K., Ikeda Y., Matsuda J., Li H.W., Tanaka I., Akiba E., Horita Z. (2018). Bulk nanocrystalline gamma magnesium hydride with low dehydrogenation temperature stabilized by plastic straining via high-pressure torsion. Scr. Mater..

[42] Kitabayashi K., Edalati K., Li H.W., Akiba E., Horita Z. (2020). Phase transformations in MgH_2_-TiH_2_ hydrogen storage system by high-pressure torsion process. Adv. Eng. Mater..

[43] Wang Q., Tang Y., Horita Z., Iikubo S. (2022). Structural and thermoelectric properties of CH_3_NH_3_SnI_3_ perovskites processed by applying high pressure with shear strain. Mater. Res. Lett..

[44] Rogl G., Ghosh S., Wang L., Bursik J., Grytsiv A., Kerber M., Bauer E., Malik R.C., Chen X.Q., Zehetbauer M. (2020). Half-Heusler alloys: Enhancement of ZT after severe plastic deformation (ultra-low thermal conductivity). Acta Mater..

[45] Edalati K., Horita Z. (2010). Application of high-pressure torsion for consolidation of ceramic powders. Scr. Mater..

[46] Razavi-Khosroshahi H., Edalati K., Emami H., Akiba E., Horita Z., Fuji M. (2017). Optical properties of nanocrystalline monoclinic Y_2_O_3_ stabilized by grain size and plastic strain effects via high-pressure torsion. Inorg. Chem..

[47] Hidalgo-Jimenez J., Wang Q., Edalati K., Cubero-Sesín J.M., Razavi-Khosroshahi H., Ikoma Y., Gutiérrez-Fallas D., Dittel-Meza F.A., Rodríguez-Rufino J.C., Fuji M. (2020). Phase transformations, vacancy formation and variations of optical and photocatalytic properties in TiO_2_-ZnO composites by high-pressure torsion. Int. J. Plast..

[48] Porz L., Klomp A.J., Fang X., Li N., Yildirim C., Detlefs C., Bruder E., Höfling M., Rheinheimer W., Patterson E.A. (2021). Dislocation-toughened ceramics. Mater. Horiz..

[49] Dong L.R., Zhang J., Li Y.Z., Gao Y.X., Wang M., Huang M.X., Wang I.S., Chen K.X. (2024). Borrowed dislocations for ductility in ceramics. Science.

[50] Yesudhas S., Levitas V.I., Lin F., Pandey K.K., Smith J.S. (2024). Unusual plastic strain-induced phase transformation phenomena in silicon. Nat. Commun..

[51] Fujita I., Edalati K., Wang Q., Arita M., Watanabe M., Munetoh S., Ishihara T., Horita Z. (2020). High-pressure torsion to induce oxygen vacancies in nanocrystals of magnesium oxide: Enhanced light absorbance, photocatalysis and significance in geology. Materialia.

[52] Razavi-Khosroshahi H., Edalati K., Arita M., Horita Z., Fuji M. (2016). Plastic strain and grain size effect on high-pressure phase transformations in nanostructured TiO_2_ ceramics. Scr. Mater..

[53] Katai M., Edalati P., Hidalgo-Jimenez J., Shundo Y., Akbay T., Ishihara T., Arita M., Fuji M., Edalati K. (2024). Black brookite rich in oxygen vacancies as an active photocatalyst for CO_2_ conversion: Experiments and first-principles calculations. J. Photochem. Photobi. A.

[54] Hidalgo-Jiménez J., Akbay T., Ishihara T., Edalati K. (2023). Understanding high photocatalytic activity of the TiO_2_ high-pressure columbite phase by experiments and first-principles calculations. J. Mater. Chem. A.

[55] Shundo Y., Ngyuen T.T., Akrami S., Edalati P., Itagoe Y., Ishihara T., Arita M., Guo Q., Fuji M., Edalati K. (2024). Oxygen vacancy-rich high-pressure rocksalt phase of zinc oxide for enhanced photocatalytic hydrogen evolution. J. Colloid. Interface Sci..

[56] Edalati K., Toh S., Ikoma Y., Horita Z. (2011). Plastic deformation and allotropic phase transformations in zirconia ceramics during high-pressure torsion. Scr. Mater..

[57] Akrami S., Murakami Y., Watanabe M., Ishihara T., Arita M., Guo Q., Fuji M., Edalati K. (2022). Enhanced CO_2_ conversion on highly-strained and oxygen-deficient BiVO_4_ photocatalyst. Chem. Eng. J..

[58] Han Y., Liu X., Zhang Q., Huang M., Li Y., Pan W., Zong P., Li L., Yang Z., Feng Y. (2022). Ultra-dense dislocations stabilized in high entropy oxide ceramics. Nat Commun..

[59] Yasui K., Hamamoto K. (2023). Theoretical upper limit of dislocation density in slightly-ductile single-crystal ceramics. J. Phys. Condens. Matter.

[60] Starink M.J., Cheng X.C., Yang S. (2013). Hardening of pure metals by high-pressure torsion: A physically based model employing volume-averaged defect evolutions. Acta Mater..

[61] Gubicza J. (2019). Lattice defects and their influence on the mechanical properties of bulk materials processed by severe plastic deformation. Mater. Trans..

[62] Ungár T., Gubicza J., Ribárik G., Borbély A. (2001). Crystallite size distribution and dislocation structure determined by diffraction profile analysis: Principles and practical application to cubic and hexagonal crystals. J. Appl. Crystallogr..

[63] Scardi P., Leoni M. (2002). Whole powder pattern modelling. Acta Crystallogr. Sect. A Found. Crystallogr..

[64] Rietveld H.M. (1969). A profile refinement method for nuclear and magnetic structures. J. Appl. Crystallogr..

[65] Lutterotti L., Scardi P. (1990). Simultaneous structure and size–strain refinement by the Rietveld method. J. Appl. Crystallogr..

[66] Edalati K., Hashiguchi Y., Pereira P.H.R., Horita Z., Langdon T.G. (2018). Effect of temperature rise on microstructural evolution during high-pressure torsion. Mater. Sci. Eng. A.

[67] Fujita I., Edalati K., Sauvage X., Horita Z. (2018). Grain growth in nanograined aluminum oxide by high-pressure torsion: Phase transformation and plastic strain effects. Scr. Mater..

[68] Edalati K., Fujita I., Takechi S., Nakashima Y., Kumano K., Razavi-Khosroshahi H., Arita M., Watanabe M., Sauvage X., Akbay T. (2019). Photocatalytic activity of aluminum oxide by oxygen vacancy generation using high-pressure torsion straining. Scr. Mater..

[69] Lutterotti L. (2000). Maud: A Rietveld analysis program designed for the internet and experiment integration. Acta Crystallogr. Sect. A Found. Crystallogr..

[70] Ungár T., Schafler E., Hanák P., Bernstorff S., Zehetbauer M. (2007). Vacancy production during plastic deformation in copper determined by in situ X-ray diffraction. Mater. Sci. Eng. A.

[71] Williamson G.K., Smallman R.E. (1956). Dislocation densities in some annealed and cold-worked metals from measurements on the X-ray Debye-Scherrer spectrum. Phil. Mag..

[72] Gay P., Hirsch P.B., Kelly A. (1953). The estimation of dislocation densities in metals from X-ray data. Acta Metall..

[73] Smallman R.E., Westmacott K.H. (1957). Stacking faults in face-centred cubic metals and alloys. Philos. Mag..

[74] Sen S., Halder S.K., Sen Gupta S.P. (1975). An x-ray line profile analysis in vacuum-evaporated silver films. J. Phys. D Appl. Phys..

[75] Chatterjee S.K., Halder S.K., Sen Gupta S.P. (1977). An X-ray diffraction study of lattice imperfections in coldworked fcc alloys: II. Coppergallium (α phase). J. Appl. Phys..

[76] Zhu J., Barber G., Sun X. (2022). Effects of isothermal temperature on dislocation density in bainite transformation of 4140 steel. Materials.

[77] Kuo H.K., Cohen J.B. (1983). Changes in residual stress, domain size and microstrain during the fatigue of AISI 1008 steel. Mater. Sci. Eng..

[78] Zhang K., Alexandrov I.V., Kilmametov A.R., Valiev R.Z., Lu K. (1997). The crystallite-size dependence of structural parameters in pure ultrafine-grained copper. J. Phys. D Appl. Phys..

[79] Valiev R.Z., Alexandrov I.V., Chiou W.A., Misra R.S., Mukherjee A.K. (1997). Comparative structural studies of nanocrystalline materials processed by different technologies. Mater. Sci. Forum.

[80] Griffiths M., Winegar J.E., Mecke J.E., Holt R.A. (1991). Determination of dislocation densities in hexagonal closed-packed metals using X-ray diffraction and transmission electron microscopy. Adv. X-Ray Anal..

[81] Popa N.C. (1998). The (hkl) dependence of diffraction-line broadening caused by strain and size for all Laue groups in Rietveld refinement. J. Appl. Cryst..

[82] Dyakonov G.S., Mironov S., Enikeev N., Semenova I.P., Valiev R.Z., Semiatin S.L. (2019). Annealing behavior of severely-deformed titanium grade 4. Mater. Sci. Eng. A.

[83] Nafikov R.K., Kulyasova O.B., Khudododova G.D., Enikeev N.A. (2023). Microstructural assessment, mechanical and corrosion properties of a Mg-Sr alloy processed by combined severe plastic deformation. Materials.

[84] Gubicza J. (2022). Comment on “Influence of prior deformation temperature on strain induced martensite formation and its effect on the tensile strengthening behaviour of type 304 SS studied by XRDLPA. Materials Science & Engineering A 826 (2021) 141960”. Mater. Sci. Eng. A.

[85] Razavi-Khosroshahi H., Edalati K., Wu J.H., Nakashima Y., Arita M., Ikoma Y., Sadakiyo M., Inagaki Y., Staykov A., Yamauchi M. (2017). High-pressure zinc oxide phase as visible-light-active photocatalyst with narrow band gap. J. Mater. Chem. A.

[86] Pippan R., Scheriau S., Taylor A., Hafok M., Hohenwarter A., Bachmaier A. (2010). Saturation of fragmentation during severe plastic deformation. Annu. Rev. Mater. Res..

[87] Levitas V.I., Javanbakht M. (2014). Phase transformations in nanograin materials under high pressure and plastic shear: Nanoscale mechanisms. Nanoscale.

[88] Nguyen T.T., Edalati K. (2024). Impact of high-pressure columbite phase of titanium dioxide (TiO_2_) on catalytic photoconversion of plastic waste and simultaneous hydrogen (H_2_) production. J. Alloys Compd..

[89] Levitas V.I. (1996). Large Deformation of Materials with Complex Rheological Properties at Normal and High Pressure.

[90] Meyers M.A., Mishra A., Benson A.J. (2006). Mechanical properties of nanocrystalline materials. Prog. Mater. Sci..

[91] Borbely A. (2022). The modified Williamson-Hall plot and dislocation density evaluation from diffraction peaks. Scr. Mater..

[92] Nguyen T.T., Edalati K. (2024). Efficient photocatalytic hydrogen production on defective and strained black bismuth (III) oxide. Int. J. Hydrogen Energy.

